# Rapid face adaptation distributes representation in inferior-temporal cortex across time and neuronal dimensions

**DOI:** 10.1038/s41598-017-01864-4

**Published:** 2017-05-10

**Authors:** Abdol-Hossein Vahabie, Mohammad-Reza A. Dehaqani, Majid Nili Ahmadabadi, Babak Nadjar Araabi, Hossein Esteky

**Affiliations:** 1 School of Cognitive Sciences, Institute for Research in Fundamental Sciences, P.O. Box 19395-5746 Tehran, Iran; 2 Cognitive Systems Lab, Control and Intelligent Processing Centre of Excellence, School of Electrical and Computer Engineering, College of Engineering, University of Tehran, P.O. Box 14395-515 Tehran, Iran; 3 Research Center for Brain and Cognitive Sciences, Shahid Beheshti University of Medical Sciences, Tehran, Iran

## Abstract

Neuronal networks of the brain adapt their information processing according to the history of stimuli. Whereas most studies have linked adaptation to repetition suppression, recurrent connections within a network and disinhibition due to adaptation predict more complex response patterns. The main questions of this study are as follows: what is the effect of the selectivity of neurons on suppression/enhancement of neural responses? What are the consequences of adaptation on information representation in neural population and the temporal structure of response patterns? We studied rapid face adaptation using spiking activities of neurons in the inferior-temporal (IT) cortex. Investigating the responses of neurons, within a wide range from negative to positive face selectivity, showed that despite the peak amplitude suppression in highly positive selective neurons, responses were enhanced in most other neurons. This enhancement can be attributed to disinhibition due to adaptation. Delayed and distributed responses were observed for positive selective neurons. Principal component analysis of the IT population responses over time revealed that repetition of face stimuli resulted in temporal decorrelation of the network activity. The contributions of the main and higher neuronal dimensions were changed under an adaptation condition, where more neuronal dimensions were used to encode repeated face stimuli.

## Introduction

In everyday life, primates are constantly exposed to time-varying stimuli with different exposure durations. The human brain encodes this sensory information in a high dimensional neuronal space using its adaptive recurrent neuronal networks^1–3^. These rapidly changing retinal images of the visual world are coded and quickly and effortlessly recognized^4, 5^. The flow of sequential sensory information changes the state of neuronal networks and plays an important role in the state-dependent processing of sensory information^6–8^. The time course and properties of sensory evoked neural responses are affected by preceding stimuli. How the continuously changing visual world affects the states of neuronal networks, and how these changes in states alter sensory neural computations are among the major questions in systems neuroscience.

Repeated presentation of stimuli results in suppression^9–11^ or enhancement^12–14^ of neural activities. These effects of adaptation on sensory neuron responses have been explained by the normalization model^13^. Changes in normalization signals suggest the importance of population-level interactions in adaptation effects, especially excitation-inhibition interplay. On the other hand, the dimensionality of representation is an important factor for understanding network information processing^15, 16^. However, how adaptation-induced changes at the population level influence high-dimensional representation is less understood.

The inferior temporal (IT) cortex is the last stage of the ventral visual pathway, which plays an important role in object and face perception^2, 4, 17–22^. High-level visual adaptation has been investigated in IT neurons^9, 11, 12, 23–26^, where most of the studies have been focused on the behavior of single cells. Meanwhile, adaptation-induced population level changes require more investigation. Category selectivity emerges early in IT neural activity. A brief stimulus presentation can result in sufficient information for object classification^2, 4, 5^. Although a few studies have investigated the effects of adaptation on IT single-cell responses within rapid timescales^27, 28^, most adaptation studies in the IT cortex use prolonged inter-stimulus intervals for adaptation, typically approximately 300 ms and longer^11, 26^. Stimulation of networks within rapid timescales may reveal novel aspects of the role of adaptation in information encoding.

Local inhibitory interneurons play an important role in shaping the responses of neurons and their selectivity^29, 30^. The responses of selective neurons are reduced under adaptation conditions^24, 31^. This reduction in response can change post-excitatory inhibition across neighboring neurons^13, 32^. Therefore, there is a possibility that disinhibition due to adaptation causes enhancement in a group of neurons in a network. Evidence of this disinhibition is rare in high-level visual areas. Furthermore, the excitation-inhibition balance is an important factor for shaping the correlated activity of neuronal pairs^33^. Therefore, changes in this inhibitory signal may change the correlation structure of a network. Moreover, the timing of excitation and inhibition in neuronal networks are different. Adaptation may change this timing and consequently change the temporal correlation of network activity, which may potentially result in temporal decorrelation. Some studies have reported no significant difference in the response latency of IT neurons under adapted and non-adapted conditions^11, 26^, while others have reported a delayed neural response due to adaptation^12, 27^. These delays can alter the relative timing of activity of neurons across a network and can thus induce substantial changes in the behavior of a network and alter the temporal structure of network activity.

Here, we investigated the impact of rapid face adaptation on the representation of face category information in IT single cells, as well as in neural population responses. We used all recorded neurons with a wide range of selectivity to test our hypotheses. We observed an enhancement in the network responses at later times due to adaptation. This enhancement was accompanied by a reduction in overall variability and decreased correlated activity in neural responses. Dimension reduction of neural mean response trajectories under adapted and non-adapted conditions showed a temporal decorrelation of network responses due to adaptation. These results summarize the network behavior during rapid face adaptation and suggest the possible strategy used by the network for handling repetition.

## Results

Comparison of network-level responses under adapted and non-adapted conditions shed light on information representation in recurrent neuronal networks. To study face adaptation across a population of neurons, we analyzed the responses of 674 IT neurons while monkeys viewed images presented in an RSVP paradigm during a passive fixation task^2^. Here, we compared the responses of the IT neural population in trials in which a face came after another face (adaptation or F-F condition) with the trials in which a face came after an inanimate image (non-adaptation or I-F condition).

**Figure 1:**
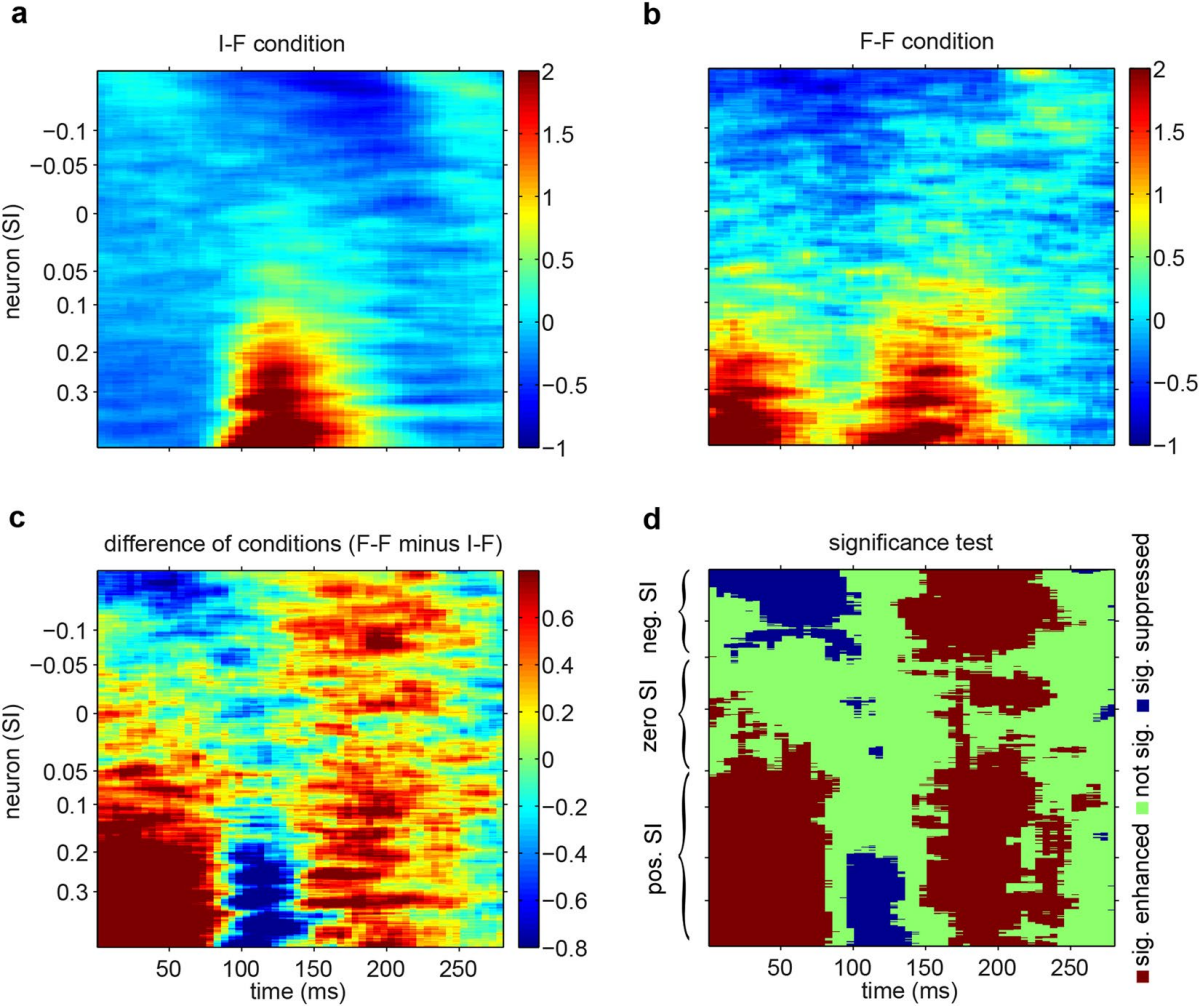
Effects of rapid face adaptation on the activity of a population of IT neurons. In (**a–d**) graphs, each row represents a neuron, which are sorted in the order of the selectivity index (SI) of the neurons, and the abscissa represents time from stimulus onset. (**a–c**) The graphs indicate the z-scored response under (**a**) the I-F condition, (**b**) the F-F condition, and (**c**) F-F minus I-F across all 674 neurons. All (**a–c**) graphs were vertically smoothed by a sliding window of 21 neurons, and the color scheme next to each graph represents the z-scored responses. (**d**) Indicates the significance test for differences across subpopulations with a sliding window of 51 neurons (paired t-test). Blue, red, and green represent significant suppression, significant enhancement, and non-significant differences, respectively, under the F-F condition compared to the I-F condition. The threshold for the p-values in (**d**) are FDR corrected for multiple comparisons. Three groupings of neurons based on their SI (pos. SI, zero SI, and neg. SI) are indicated on the vertical axis of (**d**).

Figure 1a,b shows the mean responses of all recorded neurons across time under both the non-adaptation and adaptation conditions, where each row represents a single neuron. The neurons were sorted based on their Selectivity Index (SI), which was defined as the mean responses to face minus mean responses to non-face divided by their sum. The responses were z-scored to compare neurons with different firing dynamic ranges. The color represents the z-scored responses. Since the responses from two monkeys were comparable, we pooled the neurons from both monkeys. Figure 1c shows the difference in responses under the F-F and I-F conditions (Fig. 1b minus Fig. 1a), where a substantial enhancement of neural activity that extended across almost the entire neural population was observed. To assess the significance of differences between neural activities under the two conditions, a sliding window with a length of 51 neurons on the SI axis and two-tailed t-test were utilized (Fig. 1d, see Methods). The difference between the conditions in response to the first stimuli at early times (Fig. 1d) showed that using ± 0.05 as a threshold for SI resulted in three reasonably distinct groups of neurons. Therefore, we divided neurons based on their SIs into the following three groups: neg. SI (SI < −0.05), zero SI (−0.05 < SI < 0.05), and pos. SI (SI > 0.05) neurons, which are illustrated on the SI axis of Fig. 1d. The z-scored responses from 150 ms to 200 ms after stimulus onset for each neuronal group were as follows: for pos. SI neurons: 0.54 ± 0.043 (I-F) vs. 1.06 ± 0.057 (F-F), for zero SI neurons: −0.06 ± 0.025 (I-F) vs. 0.19 ± 0.051 (F-F), for neg. SI neurons: −0.52 ± 0.024 (I-F) vs. −0.12 ± 0.043 (F-F) (mean ± SEM, paired t-test, p < 1e-19, p < 1e-4, and p < 1e-15, respectively). A significant suppressive epoch was observed for highly selective pos. SI neurons approximately the within the 100–150 ms time window (1.35 ± 0.058 (I-F) vs. 1.01 ± 0.057 (F-F), mean ± SEM across all pos. SI neurons, paired t-test, p < 1e-7). This effect was at least partially due to delayed evoked responses under the F-F condition.

**Figure 2:**
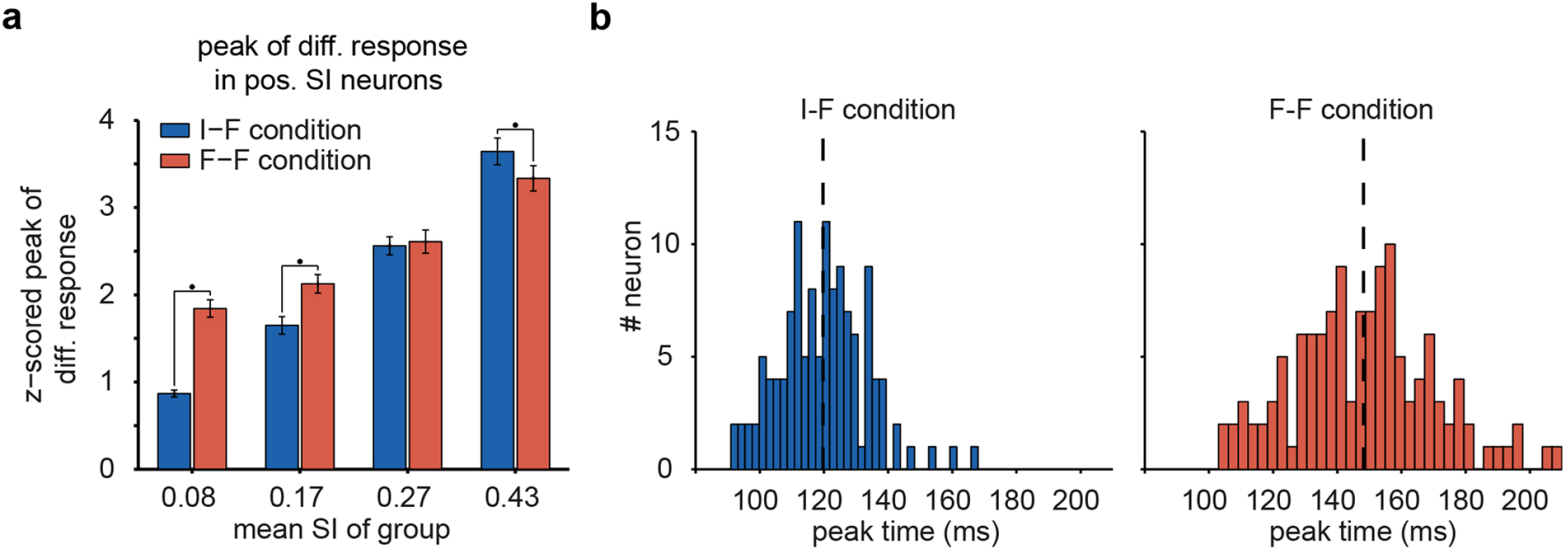
Peak value and peak time of pos. SI neurons under adaptation and non-adaptation conditions. (**a**) Peak value of the differential responses of pos. SI neurons that are divided into four equal-sized groups. The stars show the significance of differences between the F-F and I-F conditions (paired t-test, p < 0.05). (**b**) Distribution of the differential response peak time under the non-adaptation (left, I-F) and the adaptation (right, F-F) conditions for pos. SI neurons, where only neurons with robust estimation of peak time were considered (see Methods). The black dashed line indicates the mean of distribution.

The amplitude of peak responses should be investigated for a complete assessment of the suppression of neural activity. Thus, we investigated the effects of delay on the observed response pattern of pos. SI neurons. First, for each condition, we calculated the differential response of pos. SI neurons, i.e., each neuron’s responses to face stimuli minus its response to all other stimuli. Then, a smoothed version of the data (with a 25 ms Gaussian kernel) was used to find the peak time between 75 ms and 250 ms after stimulus onset. Figure 2a shows the difference between values at peak times under the two conditions for pos. SI neurons. The pos. SI neurons were divided into four groups of equal size, where the peak value was suppressed in highly selective neurons and did not change in mid-selective neurons, while the peak value was enhanced in weakly selective neurons. In other words, there was a continuum of suppression and enhancement across the pos. SI range.

The estimation of peak time for neurons with wide or flat peak responses may be less accurate. To obtain a more reliable comparison, we opted for a more conservative approach. We removed neurons with less accurate peak time estimates and obtained the peak time distributions using 126 neurons with more accurate peak time estimates (see Methods).

The peak times of the pos. SI neurons were significantly delayed on average by approximately 28 ms under the F-F condition (peak time: 119.7 ± 1.2 ms (I-F) vs. 148.1 ± 1.9 ms (F-F), mean ± SEM, paired t-test, p < 1e-10) and were more widely distributed across time. In other words, the variance of the peak times of pos. SI neurons was higher under the adaptation condition (Fig. 2b; χ^2^ variance test, p < 1e-5, F_(125,125)_: 2.47). Similar results were yielded by including the peak time data of all pos. SI neurons. In sum, the delayed response may partially explain the suppression and enhancement pattern are depicted in Fig. 1d but only for a subset of the highly positive selective neurons. In addition, the widely distributed peak times suggested distributed coding across time under the adaptation condition.

**Figure 3:**
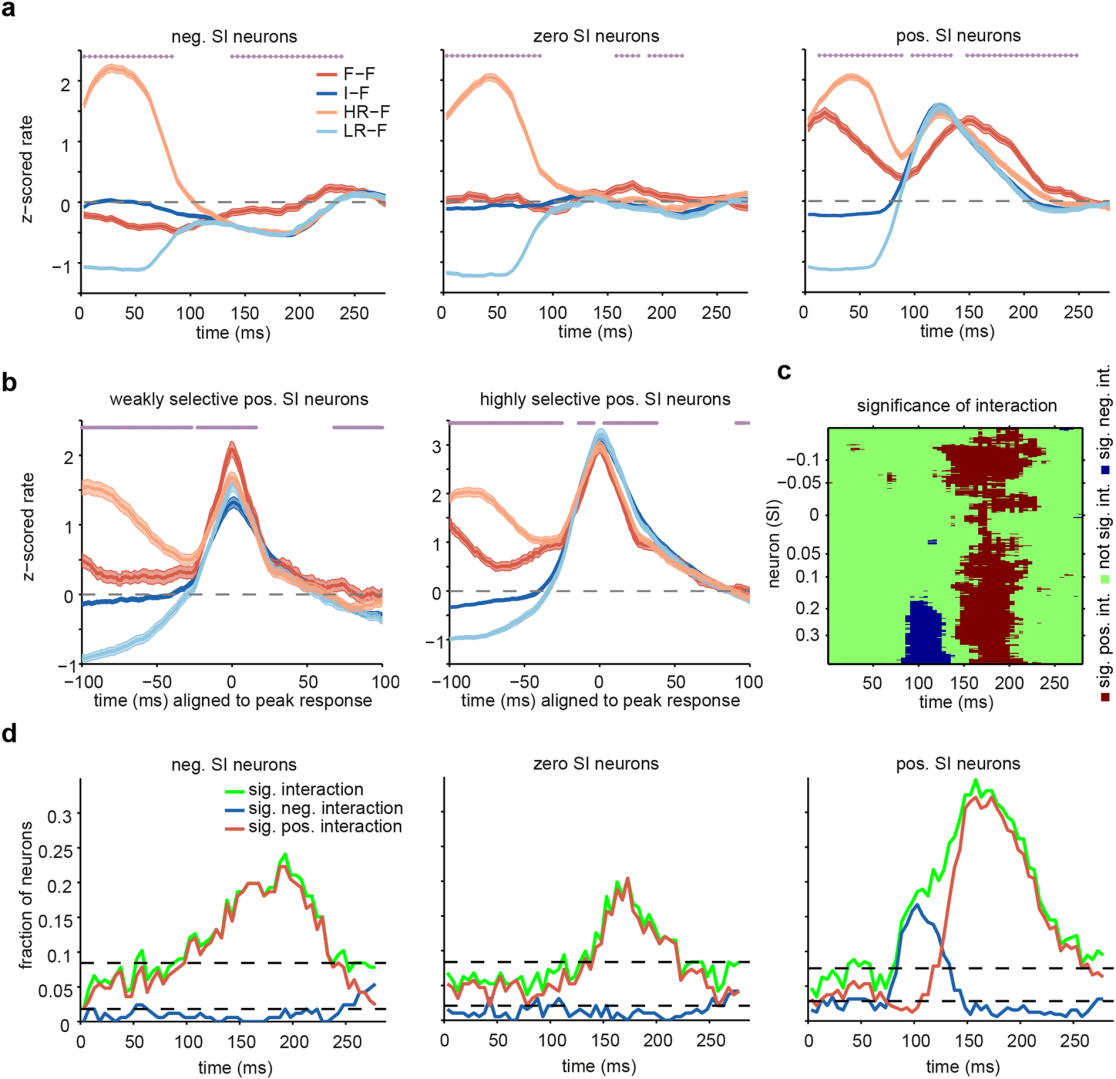
Effect of preceding stimulus rate and interaction of preceding and current stimuli on the activity of a population of IT neurons under the I-F and F-F conditions. (**a**) The time course of the averaged z-scored responses of three groups of neurons, which were defined based on the following SIs: neg. SI (SI < −0.05), zero SI (0.05 < SI < 0.05) and pos. SI (SI > 0.05) neurons, which are shown from left to right. Each time course represents the response to face stimuli with different preceding stimuli: preceded by face (F-F, red), inanimate (I-F, blue), high rate (HR-F, pale red), and low rate (LR-F, pale blue). The stars show the significance of a paired t-test between the F-F condition and all other conditions (p < 0.05). (**b**) The z-scored responses of weakly and highly selective pos. SI neurons are aligned to their peak times. Weakly and highly selective neurons were obtained by dividing pos. SI neurons into two groups of equal size. The stars show the significant difference between the F-F and I-F conditions (paired t-test, p < 0.05). (**c**) The significance pattern of interaction across a 51 neuron subpopulation estimated by a generalized linear model (GLM). Red: positive, blue: negative and green: non-significant interaction. Similar to Fig. 1d, each row represents a 51 neuron subpopulation, and the value on the ordinate is the average of SIs for those neurons. The threshold for p-values is FDR corrected for multiple comparisons. (**d**) The fraction of neurons that show significant interaction between preceding and current stimuli at each time point are shown for the three following neuronal groups: neg. SI, zero SI, and pos. SI neurons, from left to right. The GLM is fitted at each time bin across trials of each neuron. The fraction of neurons with positive interaction (red) and negative interaction (blue) are shown separately, while the total fraction is indicated in green. The black dashed lines show a 95% confidence interval for the chance level computed by a binomial test (p = 0.05).

The observed difference between the responses under the I-F and F-F conditions depicted in Fig. 1d was the result of face repetition. However, it may also be due to continued responses to the first stimulus and contamination of the response to the second face stimulus by the first. This type of contamination should be independent of the category (face or inanimate). We examined and ruled out this possibility with two additional analyses. In the first analysis, we selected the trials with face stimuli as the current stimuli and high-responsive (HR) or low-responsive (LR) non-face stimuli as the preceding stimuli. To select HR and LR stimuli, for each neuron, stimuli were ranked based on their mean responses, and the top/bottom 15% were selected as high/low responsive stimuli, respectively (see Methods). Evoked responses to faces preceded by inanimate stimulus (I-F condition) as well as LR/HR non-face stimuli (LR-F/HR-F conditions) were considerably different from those responses under the F-F condition (Fig. 3a). These results showed that firing rate (i.e., rate history) alone was not responsible for the observed face adaptation, i.e., the observed effect was face specific. To explore the time-course of neural responses around peak times, we divided the pos. SI neurons into two groups of equal sizes. Then, z-scored responses for these two groups were aligned at the peak times. The suppression of response under the F-F and HR-F conditions in the highly selective pos. SI neurons and the enhancement of responses under the F-F condition in the weakly selective pos. SI neurons are shown in the peak time-aligned responses (Fig. 3b).

In the second analysis, we tested the impact of the first stimulus on the second. We examined the interaction between preceding and current stimuli using all four of the possible conditions (F-F, I-F, F-I, I-I; see Methods). This analysis examined whether the observed difference between responses in Fig. 1d depended on the presence of a face in both the preceding and current stimuli. To this end, we used generalized linear model (GLM) analysis to quantify the effect of the interaction between the first and second face stimuli under the four conditions at the level of subpopulations of neurons (Fig. 3c) and trials (Fig. 3d). We used a GLM with a Gaussian distribution assumption on the mean responses of groups of 51 neurons (see Methods). After using false discovery rate (FDR) for multiple comparisons correction, the direction of the interaction was determined by the sign of the interaction coefficients. The interaction pattern in Fig. 3c is similar to the pattern in Fig. 1d.

To test the temporal dynamics of the interaction at the trial level, a GLM with a Poisson distribution assumption was applied to neural responses with 25 ms sliding windows. For each 25 ms time window, the number of neurons with a significant interaction between the preceding and current stimuli was determined. The positivity and negativity of the interactions were determined by the sign of the interaction coefficients. Positive/negative values indicated that face repetition resulted in higher/lower responses to the second face compared to the expected sum of the responses to the first and second face stimuli at each time window.

The proportions of neurons with significant interaction across time are shown in Fig. 3d for the three groups of neurons. There were two types of the interaction profile in the pos. SI neurons. Highly selective neurons exhibited a peak of negative interaction at approximately 100 ms, while neurons with lower face selectivity exhibited a peak of positive interaction at approximately 150 ms. No significant negative interaction was observed for neg. SI and zero SI neurons at the early phase (Fig. 3d). In contrast, a significant positive interaction was observed across all neuronal groups at the later phase of responses. All peaks above the broken line are highly significant (binomial test, p < 0.05).

### Consequences of changes in rate pattern due to adaptation

The observed rate pattern may change the processing of face information across the IT cortex. To study this issue, we first examined the changes in spike count variability and correlated variability across the network. Both variability and correlation changes between neurons alter the information processing capacity of the network. In the second analysis, we used Principal component analysis (PCA) to investigate the population response trajectory in a high-dimensional neuronal space.

**Figure 4:**
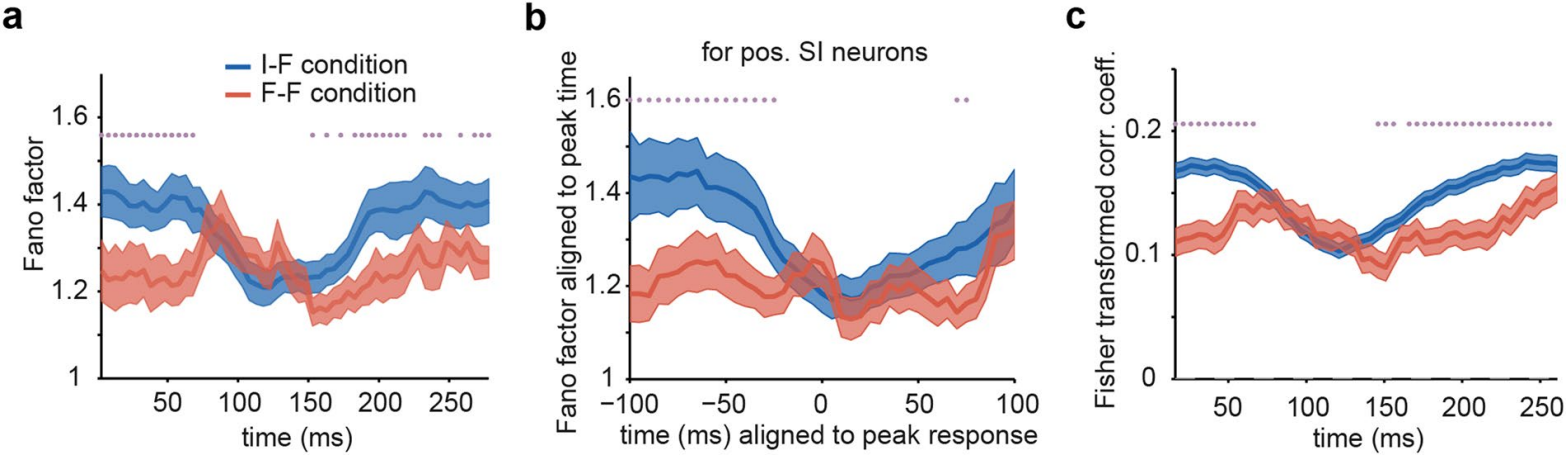
Variability of spiking activity and correlation of neuronal responses under the I-F and F-F conditions. (**a**) The time courses of the Fano factor across all neurons. The Fano factor at each time point was computed by the slope of the regression line between the mean and variance of response across all neurons. The stars show significant differences between two conditions, which were assessed by a one-tailed z-test. (**b**) The Fano factor of pos. SI neurons aligned to the peak time of neuronal responses. The responses of the neurons were aligned to the peak time of their differential response, and the mean-matched Fano factor was calculated similarly to that shown in (**a**). (**c**) The time-course of correlation between simultaneously recorded pairs of neurons, which was calculated using z-scored responses. To avoid state-dependent correlations, z-scoring was performed based on neighboring trials. The stars show the significance of differences between two conditions (paired t-test, p < 0.05). In all graphs, blue and red curves indicate the non-adaptation (I-F) and adaptation (F-F) conditions, respectively. Shaded areas represent SEM.

Neurons responded to identical stimuli in different trials with variable rates. The Fano factor is a measure of noise-to-signal ratio that quantifies neuronal variability^34, 35^. To explore the effect of adaptation on the reliability of stimulus encoding in the IT cortex, the Fano factor was computed for the IT neural population over time (see Methods). During the late responses, the Fano factor was lower under the F-F condition compared with the I-F condition (one-tailed z-test, p < 0.05; Fig. 4a). The observed reduction cannot be an artifact of rate difference because we mean-matched the Fano factor. The significance of the mean-matched Fano factor difference would have been marginal if we had used a two-tailed test. The Fano factors of the adaptation and non-adaptation conditions diverged at approximately 150 ms, which was consistent with the time of rate enhancement in the later phase of the response (Figs 1d and 3c). The observed difference in variability may be due to a difference in timing between the F-F and I-F conditions. Delayed and distributed responses played an important role in the emergence of the difference as well. When we aligned the responses of pos. SI neurons to neuron peak times and computed the Fano factor (Fig. 4b), the significant differences at many time points were lost; however, the trend of lower variability have been preserved in the aligned Fano factor.

The correlated activity of neurons can change the information content in a neural population^36^. We examined and compared the network behavior under the adaptation and non-adaptation conditions by measuring the Pearson correlation between spike counts of 377 simultaneously recorded pairs of neurons. The correlation between two neurons was computed on the z-scored responses, where neighboring trials were utilized for z-scoring^37^. This z-scoring scheme helped us avoid possible contamination due to brain state and simultaneous fluctuations. The face was considered the signal, and the measured correlation was considered the noise correlation at the level of the face category.

The average of correlation coefficients across all pairs of neurons showed a significantly lower correlated activity under the adaptation condition (paired t-test, p < 0.05, Fig. 4c). The correlation curves of the F-F and I-F conditions were dissociated at approximately 150 ms, which was consistent with the time of the rate enhancement in the later phase (Fig. 1d) and the time of the variability reduction (Fig. 4a). The variability and correlation were due to both trial by trial and image by image variabilities. We could not disentangle these variabilities because of the low number of trials for each individual image in the F-F condition.

### Signature of face adaptation in neural response trajectories in the high-dimensional neural space of the IT cortex

**Figure 5:**
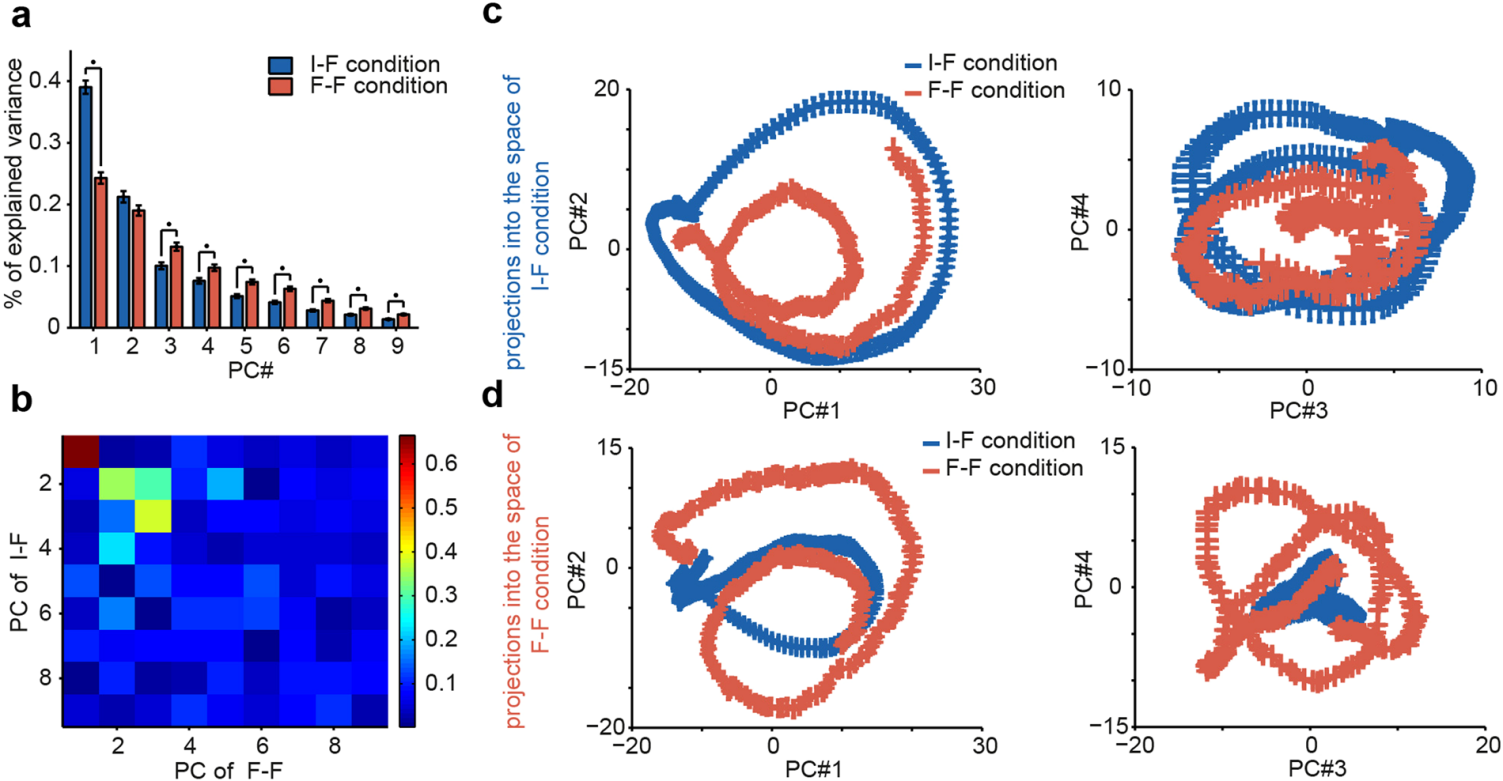
Principal components (PCs) of the network and difference between the adaptation and non-adaptation conditions. PCs—eigenvectors—of the network’s mean activity under the non-adaptation (blue) and adaptation (red) conditions are extracted separately by PCA. (**a**) The percentage of explained variance by each PC in two conditions. The stars show the significance of differences using a z-test. (**b**) Rotation of PCs in two conditions are quantified by the absolute value of their pairwise dot product (cosine of the angle between eigenvectors). The rows and columns are representative of the eigenvectors of the non-adaptation and adaptation conditions, respectively. (**c**,**d**) The projection of the network response trajectory under the non-adaptation and adaptation conditions into the first four PCs of the two PC spaces: (**c**) the projections into the space constructed from the non-adaptation responses, (**d**) the projections into the space constructed from the adaptation responses. Red and blue curves show the projections of adaptation and non-adaptation responses into the spaces, respectively. In (**c,d**) the graphs on the left show the projections into PC1 vs. PC2, and the graphs on the right are the projections into PC3 vs. PC4. The error bars indicate SEM.

Thus far, our results have shown that repeated exposure to the face category in a rapidly changing visual environment may alter the encoding capacity at the neural level. Our previous observations have indicated the presence of category encoding at the neural population level^2, 38^. To further explore the impact of adaptation on face representation, we applied PCA to the mean response trajectories of neurons. Using PCA helped us examine the coordinated activities of neurons under the I-F and F-F conditions. For each condition, eigenvalues of PCA quantified the contribution of the different neural directions to explain the trajectory of the response. Eigenvectors or Principal Components (PC’s) were sorted in the order of eigenvalues, from large to small. The first PC had a significantly lower contribution to overall explained variance in the adaptation condition compared to the non-adaptation condition, while the third and higher PCs had a significantly larger contribution in the adaptation condition (z-test, p < 0.05, Fig. 5a). If we had used eigenvalues instead of explained variances, we would have obtained similar results. These results indicated a more distributed face representation across the IT neural population under the adaptation condition.

To investigate whether the directions of the PCs in two conditions were aligned, we computed the inner products of eigenvectors, i.e., the cosine of angles between eigenvectors (see Methods). These values indicated an overlap between the neurons that were important in defining the directions. Interestingly, the PCs were rotated in the F-F condition relative to the I-F condition (Fig. 5b). The first three eigenvectors of the two conditions were relatively aligned, while the higher order PCs were approximately orthogonal. The observed rotation in Fig. 5b indicated a modification of the role of the IT neurons in face representation due to adaptation.

Thus far, in the PCA analysis, two separate PCA spaces for the F-F and I-F conditions were obtained. To further explore the network dynamics, we projected the response matrix of each condition into both PCA spaces and obtained projected responses across time. The projected responses had oscillatory patterns. The oscillation frequencies were a function of the rank of the PCs, where the lowest frequency was observed for the first PC. Oscillatory activity over the time course of projections resulted in a rotatory pattern for the corresponding trajectories in neural spaces, Fig. 5c,d. The radius and number of rotations indicated the amplitude and frequency of oscillation, respectively.

Our PCA analysis was similar to a spectral analysis in high-dimensional space and decomposed the network activity into its oscillatory components. Adaptation reduced the low-frequency components, whereas it enhanced the higher-frequency components. Thus, the mean activity of the network under the F-F condition was more temporally decorrelated than that under the I-F condition. In our temporal PCA analysis, we used mean responses at different time points and utilized temporal correlations to extract PCs. Therefore, the observed decorrelation in the explained variance pattern for the adaptation condition was an indicator of temporal decorrelation of the network activity. Due to adaptation-induced temporal decorrelation, the network used different neural dimensions at different time points. The trajectory of the response traveled in more neural dimensions under the adaptation condition.

**Figure 6:**
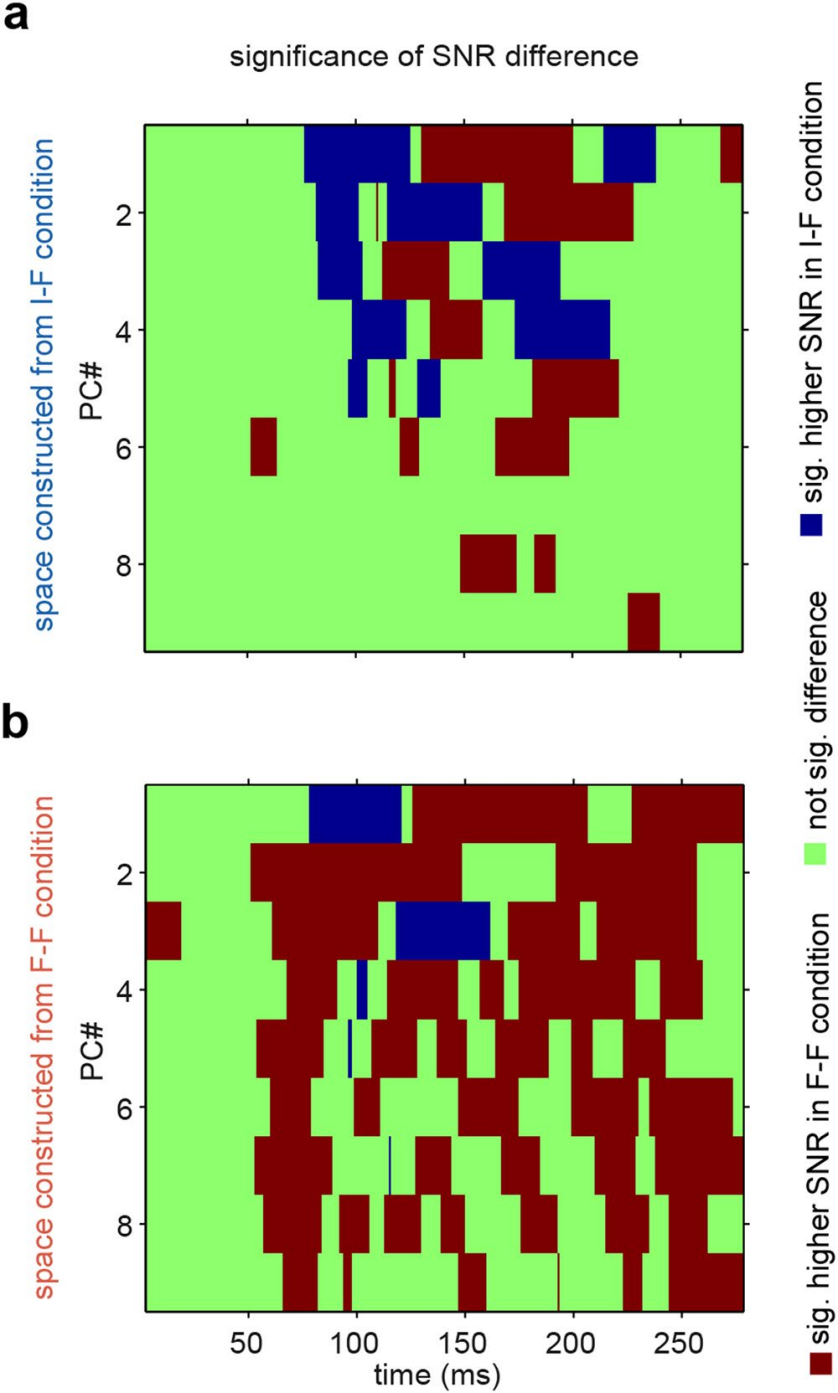
SNR between face and all other animal images. The SNR was calculated for each direction of the two spaces and for responses under each condition and then significance of difference between the responses under the F-F condition and responses under the I-F condition are shown for (**a**) the space constructed from the I-F condition, and (**b**) the space constructed from the F-F condition. Significance was assessed using a one-sample z-test (p < 0.01, FDR corrected, q < 0.01).

To examine the information content of each direction at each space, we used a signal-to-noise ratio (SNR), defined as the ratio of between-class to within-class variances at each direction of each space (see Methods). The SNR was calculated between faces and all other animate images. Figure 6 depicts the pattern of significant differences between responses under the F-F and I-F conditions at each space (one-sample z-test, p < 0.01, FDR corrected, q < 0.01). In the I-F condition space (Fig. 6a), the F-F condition responses had significantly lower SNRs at some time points of the first four PCs. While at other times and especially in higher dimensions, the F-F condition responses had significantly higher SNRs. In the F-F condition space (Fig. 6b), a significantly lower SNR was observed in the responses under the F-F condition but only at early times in the first and third dimensions. Meanwhile, the F-F condition responses had significantly higher SNRs over a wide range of time points and almost all directions. Thus, we can state that adaptation rotated the space in directions in which information was widely distributed across time and neuronal dimensions.

## Discussion

We examined the impact of face adaptation on face representation at both single-cell and network levels in the IT cortex. Neural data from the adaptation (F-F) and non-adaptation (I-F) trials were compared. We found that the evoked responses of pos. SI neurons to faces were delayed under the F-F condition compared to those under the I-F condition. Adaptation enhanced the response of the network in later parts of the evoked neural activity. The observed late enhancement of activity was accompanied by a reduction in spike count variability and correlated activity. The IT neural population code was altered by face adaptation; the contribution and significance of the main components—defined by temporal PCA—were decreased and those of higher order components were increased under the F-F condition. Therefore, a face in the F-F condition was represented in a more distributed manner, which required more neural dimensions (PCs), compared with a face in the I-F condition. The IT neural population activities were temporally decorrelated.

One limitation of our data is the lower number of trials in the adaptation condition, but the same conclusions were obtained when the trial numbers were equalized using resampling. Although equalizing the trial numbers might decrease the effect size in some cases, the different trial numbers did not alter any of our results. Our data have enough trials for categorical analysis of face adaptation; however, the number of trials for individual faces was not enough for more detailed analysis. Therefore, we limited our analysis to the level of the face category.

Suppressive effects of adaptation have been described by the following neural level models: suppression due to fatigue (after-hyperpolarization), short-term plasticity (STP in synaptic level) or input fatigue, facilitation model, sharpening model, synchronization hypothesis and predictive coding^10, 14, 39–42^. Our results provided evidence in favor of the input fatigue model and in opposition to the facilitation model. The reported adaptation effect in our data was not due to the firing rate of neurons in response to the preceding stimulus and was face specific. The fatigue model alone cannot describe our results. Face specificity suggests an input channel-specific mechanism for the observed effects. The observed suppressive effects on highly selective pos. SI neurons were consistent with other studies on the suppressive effects of adaptation, which have also shown the specificity of the results to the similarity of preceding and current stimuli—not the firing rate of neurons in response to the preceding stimuli^9, 26^. In particular, it has been shown that input fatigue and/or synaptic level STP were more consistent with the neural data^11^.

Reported delayed response contradicts the facilitation model. Delayed response can be described by both after-hyperpolarization and synaptic dynamics; however, the duration of the mean delay was shorter when a face was preceded by the high-rate stimuli (HR-F vs. F-F). Therefore, our data are more in favor of synaptic delay. The amount of delay between the F-F and I-F conditions is consistent with the findings of Perrett *et al*.^27^. The authors reported a delayed and decayed response for IT neurons when preceding and current stimuli were similar.

We cannot rule out top-down effects at the later times. However, in our data, the rapid presentation paradigm and the lack of late enhancement in the case of intervening stimuli were not in favor of top-down effects. The expectation of face stimuli can enhance the response of neurons^43^. Moreover, it has been shown that the responses of IT neurons under expectation and surprise conditions are the same^44, 45^. However, in our study, the probability of face stimuli was smaller than that of non-face stimuli, and the probabilities of the first and second face were similar; therefore, expectation and surprise are unlikely to describe the observed enhancement.

Two factors may contribute to the observed temporal decorrelation. First, the peak of responses in pos. SI neurons were more distributed across time under the F-F condition. Therefore, they were more aligned under the I-F condition and more correlated. Second, the valley of the responses in neg. SI neurons was missing under the F-F condition. This result was due to the enhancement of response in neg. SI neurons and was likely due to the reduction of inhibition to these neurons.

Indeed, the minimum time of response in highly negative selective neurons was later than the peak time of highly positive selective neurons. This result reinforces the view that negative selectivity can be constructed by the inhibition from pos. SI neurons. The observed adaptation-induced enhancement in the responses of neg. SI neurons could be caused by the reduction in recurrent inhibition. Recurrent inhibition is one possible mechanism for implementing the normalization signal^46^. One prediction of the normalization model for adaptation is that if adaptation reduces the normalization signal, there should be an enhancement in response in at least some groups of neurons. Reductions in post-excitatory inhibition in the adaptation of V1^32^ and analyses of adaptation effects with the normalization model^13, 47^ are consistent with this observation. On the other hand, the normalization model has also been hypothesized as the building block of subspace untangling in the visual pathway^3^. A normalization signal causes a smoother representation of an object. Thus, increased dimensionality is expected, if the normalization signal under the adaptation condition is reduced. Our results are consistent with the predictions of the normalization model of adaptation^13^; however, we have not ruled out other possible, more complex explanations.

Dissociation between adaptation and non-adaptation conditions in terms of noise correlation and variability as well as late rate enhancement all start at approximately 150 ms after stimulus onset. In several studies, this particular time has been associated with the maximal shape selectivity and peak of global information representation as well as other types of selectivity in the inputs of the IT cortex^48^. Around this time, global information begins to drop, while detailed information begins to rise^49–52^. It has been shown that linear and nonlinear parts of shape coding are processed via feed-forward and recurrent processing, respectively; Recurrent nonlinear processing dominates feed-forward linear processing at approximately 150 ms^51^. Therefore, the observed late enhancement may, at least partially, be associated with changes in recurrent processing under the adaptation condition. This recurrent processing may involve the normalization signal as its main part. Comparing our results to these findings suggested the possible following role for adaptation: enhancement of detail processing.

Because of the limited number of trials for individual faces in the adaptation condition, we did not have enough statistical power to analyze SNRs at the identity level. Nevertheless, some hints from data have encouraged us to propose a normative conjecture that describes what occurs during the adaptation, which can be tested in future works. During the first presentation of a stimulus from a category, processing of global information is more likely to occur in the main dimensions and processing of detailed information is performed across the whole network, mainly in higher dimensions. The arrival of another stimulus from the same category does not trigger global information processing in the main dimensions as much as the first presentation, while it boosts detailed information processing and presentation in higher order dimensions. In other words, during the processing of the first stimulus, the network goes to a state where the excitability of its main dimensions is decreased for upcoming similar stimuli, while higher order dimensions become more represented. If adaptation increases the dimensionality of data, higher dimensions can enhance the detailed processing. Both temporal decorrelation and correlation reduction hint at an increase in dimensionality. Thus, we propose that the changes in representation and increase in dimensionality during rapid adaptation can be beneficial for the enhancement of detail processing. This conjecture is in line with previously proposed hypotheses on the benefits of adaptation, which include improved discrimination, efficient coding, and saliency improvement^10, 13, 53, 54^.

In sum, we found that adaptation changes the pattern of activity in the IT cortex and alters its high-dimensional representation. The temporal structure of the pattern of network activity is an important factor for understanding the consequences of adaptation in cognition and behavior.

## Methods

### Paradigm and recording

The neural data used here have been previously reported^2^. In brief, over 1000 colorful photographs of various animate and inanimate objects were presented to two male rhesus macaque monkeys on a gray background in a rapid serial visual presentation (RSVP) paradigm. The duration of each stimulus was 105 ms without any inter-stimulus blank interval. The images were shown in a 7-degree window at the center of a CRT screen. The fixation window was ±2 degrees at the center of the monitor screen. The responses of 674 single neurons of IT cortex were recorded as the monkeys passively viewed the presented images. All experimental procedures were in accordance with the guidelines of the National Institutes of Health and the Iranian Society for Physiology and Pharmacology and were approved by the animal care and use committee of the Institute for Research in Fundamental Sciences (09-13-61012002).

### Data analysis

To explore the effect of sequential face presentation, we divided the face trials into the following two groups: trials that were preceded by another face (adaptation or F-F condition) and trials that were preceded by an inanimate stimulus (non-adaptation or I-F condition). The face trials included both monkey and human faces. The inanimate images mainly included objects, scenes, simple shapes and plants. Non-primate faces and non-face animate images were excluded from both the face and inanimate stimulus sets. The sample size was different between the F-F and I-F conditions (45.4 ± 0.8 and 348.4 ± 2.7, mean ± SEM across trials, respectively). In the following analyses, our conclusions remained intact after sample size matching, i.e., after trial numbers were matched between the conditions using resampling (for more details, see below).

Selectivity of neurons to faces was quantified using the following Selectivity Index (SI)^55^.

1 $$SI=\frac{\bar{R}(face)-\bar{R}(non\_face)}{\bar{R}(face)+\bar{R}(non\_face)}$$

where $\bar{R}(face)$ and $\bar{R}(non\_face)$ are the average responses to face and non-face trials, respectively. Here, the response is the spike count in the time window of 75 to 200 ms after the stimulus onset.

Neurons were further categorized into three following subgroups based on their SI: 1) negative selectivity (neg. SI) SI < −0.05; 2) zero selectivity (zero SI) −0.05 < SI < 0.05; and 3) positive selectivity (pos. SI) SI > 0.05. Alternatively, the negative selectivity, zero selectivity and positive selectivity groups can be referred to as the non-face selective, non-selective, and face-selective groups, respectively. The total numbers of the neg., zero, and pos. SI neurons were 166, 191, and 317, respectively. The results were not sensitive to the threshold value for neuronal grouping.

Spike counts at each trial were computed using a 25 ms sliding window with 5 ms steps. To compare neurons with different dynamic ranges, z-scored values of spike counts were used. The mean and standard deviations for z-scoring were computed by using the mean responses to each image from 75 to 180 ms.

To examine the statistical significance of the differences in responses under the F-F and I-F conditions over time, we employed a conservative method. If we had applied the test on each neuronal group, including all neurons in the group, the significant difference would have been more evident. However, to show the pattern of difference across the neural population and time, we used the following method. We sorted neurons based on their SI values and then selected the first 51 neurons, computed a two-tailed paired t-test for the z-scored mean responses under the I-F condition vs. the F-F condition and repeated the same procedure by moving one neuron down the SI list until all neurons were tested in the sliding groups of 51 units. After obtaining all p-values for all time bins and all subpopulations, FDR was used to correct for multiple comparisons (q < 0.1). It has been argued that q < 0.1 is also a reasonable value for multiple comparisons^56^. In addition, the large clusters of significant areas that we observed were unlikely to be due to multiple comparisons. Considering the cluster size in this study, q < 0.1 better depicted the differences between the conditions. If we used q < 0.05 for FDR, the conclusions remained intact with a small reduction in the significant areas.

To compare the peak value of neural responses and their timing in pos. SI neurons, spikes were counted in a 25 ms sliding window with a step of 1 ms. Smoothing by Gaussian kernel (σ = 25 ms) was also applied for the determination of peak time. To avoid any artificial peak due to the firing rate of the preceding stimulus, we estimated the peak of response from the differential response to face, which is computed as the average response to faces minus the average response to all other stimuli at a particular preceding stimulus condition, i.e.,

2 $$\begin{array}{rcl}Diff\_{\bar{R}}_{t}(face|{\rm{pre}}\_category) & = & \bar{R}(face|{\rm{pre}}\_category)\\  &  & -\,{\bar{R}}_{t}(all\_other\_stimuli|{\rm{pre}}\_category)\end{array}$$

where ${\rm{pre}}\_category$ could be any inanimate or face as the preceding stimulus, and t refers to time. The peak values were determined between 75 ms and 250 ms after stimulus onset.

The estimation of peak time was less accurate for some neurons due to some factors, such as low spiking activity or sustained flat evoked responses without a sharp peak. To avoid these miscalculations, we applied a more conservative approach for the comparison of peak times and eliminated neurons with less accurate estimations of peak time. Preserving all neurons did not change the results. The standard error of the mean (SEM) of peak time estimation was computed to select neurons with more accurate and robust estimations of peak time. To this end, we resampled with replacement from trials of each condition with the same number of trials as the adaptation condition and calculated the peak time. This procedure was repeated 300 times. Neurons with peak time SEMs less than 25 ms (length of the sliding window) were selected for peak time analysis. Distributions of the peak times of the selected neurons under the two conditions had different means and variances. Comparison of mean differences was conducted using a paired t-test, while the significance of variance differences was checked with a two-tailed χ^2^ variance test.

To see how the firing rate history modulated neural responses to face stimuli, for each neuron, we ranked the stimuli based on their mean evoked responses (75–200 ms). The upper 15th percentile of ranked images was defined as high-responsive (HR) images, while the lower 15th percentile of ranked images was defined as low-responsive (LR) images. We excluded the face stimuli from both the HR and LR groups. We analyzed and compared the responses to faces when the preceding stimuli were from these high/low responsive groups. We compared the responses under the high-face (HR-F) and low-face (LR-F) conditions with those under the adaptation condition. A two-tailed paired t-test was used for the investigation of significant differences.

We examined the interaction between two consecutive stimuli at the level of the trial as well as the subpopulation levels. At the trial level, a GLM with a Poisson distribution was applied. First and second covariates were the current and preceding stimuli, respectively, whereas face and inanimate were labeled one/zero. Interaction of covariates was also added to the model. The dependent variable was the number of spikes at each time bin. A binomial test was utilized to check the significance of the fraction of neurons with significant interaction across the three groups of neurons (see above). A positive/negative sign of interaction coefficient was interpreted as enhancement/suppression. At the subpopulation level, we grouped neurons into 51 units based on the SI values and applied the GLM with a Gaussian distribution to the mean responses of the neurons. These values were calculated for four pairs of current and preceding stimuli depending on the following order of the face (F) and inanimate (I) stimuli: F-F, F-I, I-F, I-I. Covariates were the same as those of the single neuron level GLM. The significance threshold for interaction was set to 0.05. The calculated p-values were FDR corrected.

The variability of the neural responses to face stimuli were quantified for both the adaptation and non-adaptation conditions. Due to the lower number of trials in the adaptation condition, for each neuron, we resampled trials (with replacement) of both conditions with the same size as the number of adaptation condition trials. Then, variance and mean of spike counts were computed for each neuron and condition in 25 ms sliding window with 5 ms steps. To calculate the Fano factor for each group of neurons, the slope of the regression line between the variance and the mean was computed at each time point^57^. We also computed the mean-matched Fano factor between conditions^35^. Briefly, after the calculation of variance and mean response of each neuron for each condition and at each time point, distributions of means were equalized between two conditions. This equalization at each specific bin was performed by randomly removing neurons from the condition with a higher number of neurons. This equalization strategy indicates that the matching procedure was applied separately for each time point. The significance test between the two conditions was assessed using a one-tailed z-test. The calculated face category variability consisted of trial by trial variability and image by image variability; therefore, it was different from the conventional trial by trial variability. The same argument is true for the following quantification of correlated activity (see below).

There were 377 pairs of simultaneously recorded neurons in our dataset. To observe the effect of adaptation on the correlation between the spiking activities of these pairs of neurons, we computed the time-course of the Pearson correlation between spike counts of each neuronal pair under both conditions. To avoid sample size bias, we repeatedly resampled trials to maintain the same number of trials in the non-adaptation condition and the adaptation condition. Here, we used a 50 ms sliding time window for the spike count to achieve more reliable correlation coefficients. Brain state alterations during recording may cause correlated activities across trials. To cancel out these possible confounding factors, we used z-scored values of the spike counts at each trial separately, while z-scoring used the mean and standard deviation of neighboring trials (400 image presentations)^37^.

3 $${Z}_{trial}=\frac{{R}_{trial}-{{\mu }}_{neighboring\_trials}}{{\sigma }_{neighboring\_trials}}$$

Then, the correlation coefficients between simultaneously recorded trials were computed using these z-scored responses. The calculated correlation coefficients did not differ significantly from the raw correlations. We used Fisher’s z-transformation to make the distribution of correlation coefficients approximately Gaussian. Then, we applied a two-tailed paired t-test to the transformed values across neurons.

To compare the trajectories of neural responses in high-dimensional neural space, we performed PCA on the mean responses of neurons under the adaptation and non-adaptation conditions. At first, mean z-scored values of neural responses at 25 ms sliding windows with steps of 1 ms were arranged in a matrix (number of neurons by number of time bins) for each condition. Then, using PCA on data from 75 ms to 250 ms, we decomposed the response matrix into its PCs. Each PC is an eigenvector of the covariance matrix, and its eigenvalue shows the contribution of that PC to the overall variance of the trajectory in neuronal space across time.

To obtain the standard errors of the eigenvalues, we ran PCA on matrices that were constructed from the same number of resampled trials as the adaptation condition. Significant differences were assessed using a z-test. To compare the spaces of the two conditions, the similarity of the eigenvectors of the two spaces were quantified by the absolute value of the dot product of the eigenvectors, i.e., the cosine of the angle between eigenvectors. Furthermore, the projection of the response matrix of each condition into the spaces of both PCs is shown. The projection of the response of population at each time point was projected on each PC’s direction. Therefore, the projection of the response matrix provided the time course of projected values at each component. Then, we plotted these values in PC1 versus PC2 and PC3 versus PC4 and connected and aligned them temporally. Our temporal PCA analysis decomposed the temporal correlation structure of the network responses because of the manner in which we constructed the covariance matrix from samples at different time points.

To quantify the amount of information at each PC direction, we used all animate images except our face category and defined the signal in each condition as the discrimination between face and animate images in those conditions, i.e., when preceded by face images (adaptation) and when preceded by inanimate images (non-adaptation). The following signal to noise ratio (SNR) at any direction in high-dimensional space and between two categories was defined as:

4 $$SNR=\frac{{({\omega }^{T}({\mu }_{A}-{\mu }_{B}))}^{2}}{{\omega }^{T}({{\rm{\Sigma }}}_{A}+{{\rm{\Sigma }}}_{B})\omega }$$

where *ω* was any direction at high dimensional space, *μ*_*X*_ was the mean of *X* category and ${{\rm{\Sigma }}}_{X}$ was its covariance matrix^58^.

Since the data consisted of different recording sessions, we do not have enough information to calculate trial by trial correlations of the neurons, so we assumed a diagonal covariance matrix for our calculation^59^. To calculate SNRs in the non-adaptation condition, we used the same number of trials as the adaptation condition by resampling 300 times without replacement. These calculated SNR values may have been biased. Bias correction was conducted by shuffling the labels of trials and subtracting the mean value of the signal to noise ratio at 300 labeled, shuffled runs. The significance of comparisons between the responses under the two conditions at each space was assessed using a one-sample z-test (p < 0.01, FDR corrected: q < 0.01).
